# Supplementary figures and images for: The multiplicity of thioredoxin systems meets the specific lifestyles of Clostridia
Source: PLoS Pathog. 2024 Feb 8;20(2):e1012001. doi: 10.1371/journal.ppat.1012001 (PMC10880999; doi:10.1371/journal.ppat.1012001)

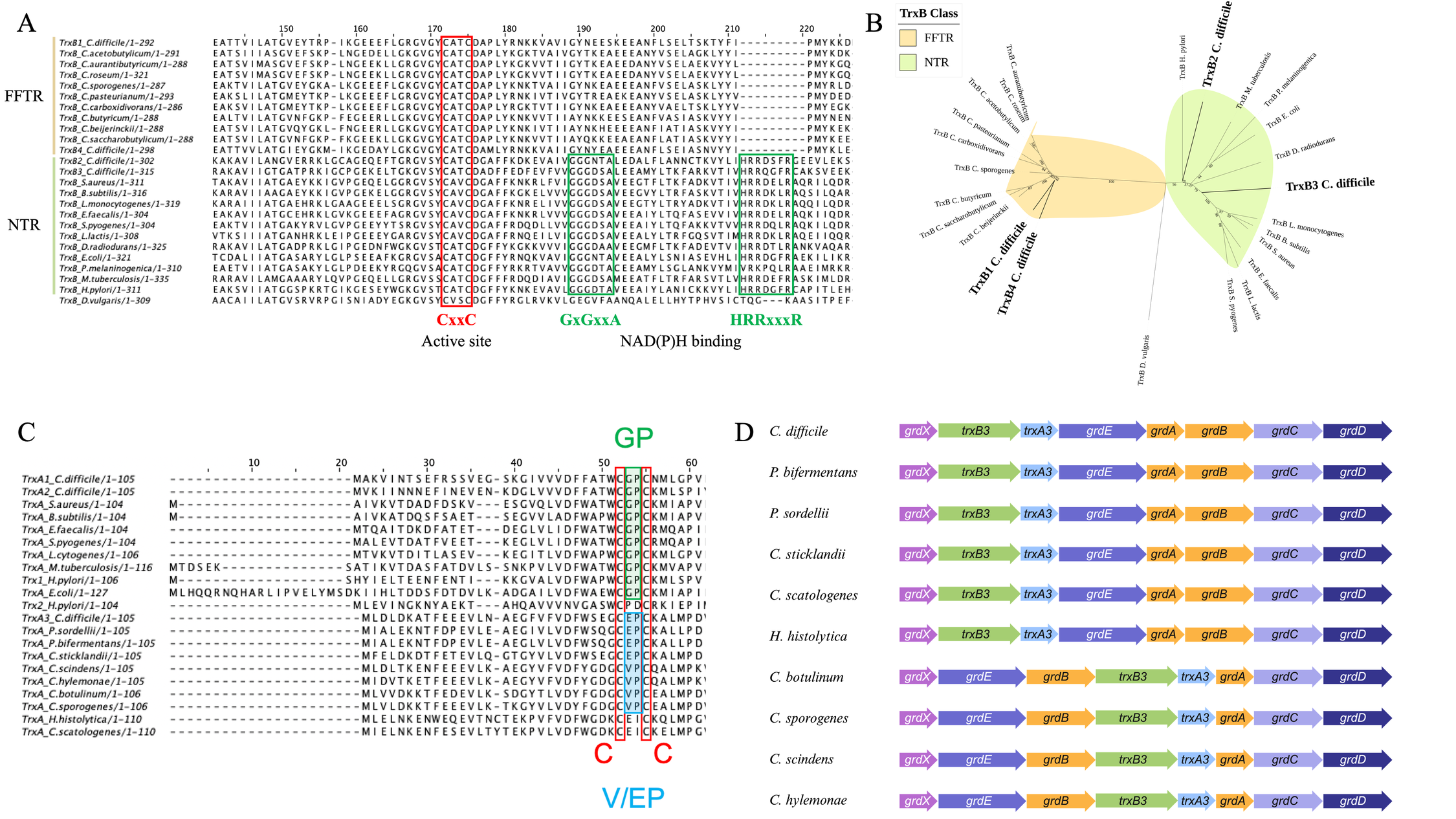

Supplement: S1 Fig — (A) Alignment of TrxB sequences from diverse bacteria. Alignment was performed using MAFFT software [112]. The CxxC active motif is indicated by a red box and the NAD(P)H binding motifs by a green box. (B) Distance tree of TrxBs was obtained using the neighbor-joining method. FFTRs cluster in orange and NTRs in green. The atypical TrxB from Desulfovibrio vulgaris [114] was used to root the tree. Bootstraps are indicated on the branches. (C) Alignment of TrxA sequences from diverse bacteria. Alignment was performed using MAFFT. CxxC active domain is indicated by a red box, the classical GP site is highlighted in green and the atypical V/EP site in blue. (D) Synteny of Clostridial grd operons. Sequence of grd operons from proteolytic Clostridia were analyzed using the MicroScope platform [57]. (TIF) [file ppat.1012001.s001.tif]

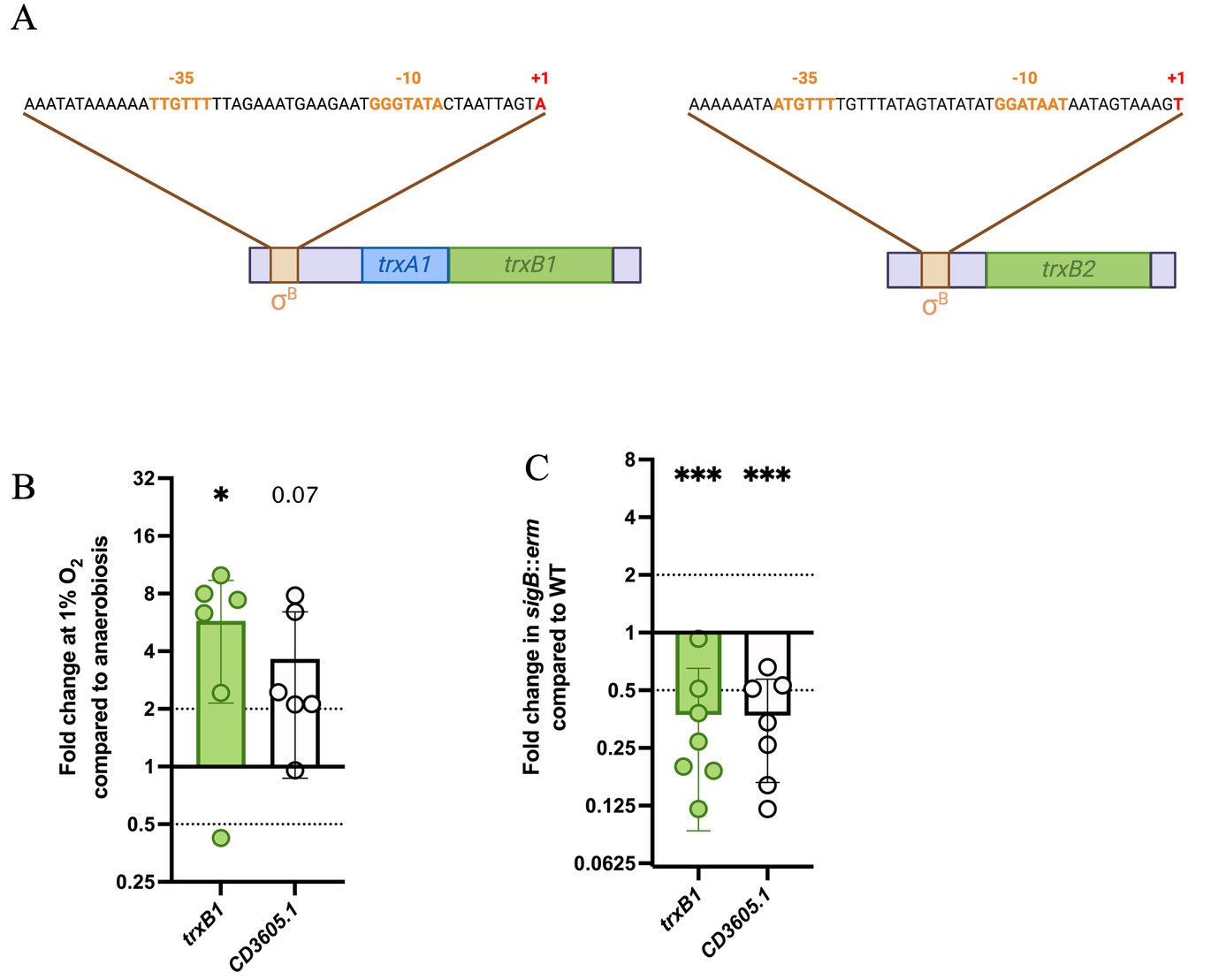

Supplement: S2 Fig — (A) Promoter identification through 5’RACE using RNA extracted from exponentially growing cells of strain 630Δerm. The TSS (+1) is indicated in red. Upstream this TSS, sB boxes are represented in orange. (B-C) Expression of the trxB1 gene and the CD3605.1 gene encoding a ferredoxin was monitored by qRT-PCR in (B) WT strain after 24 h of growth in TY medium in anaerobiosis or at 1% O2 or in (C) WT strain and sigB mutant after 4.5 h of growth in TY. Experiments were performed in at least 6 biological replicates. Mean and SD are shown. One sample t-tests were used with comparison of the fold change to 1. *: p- value<0.05, ** <0.01, *** <0.001. (TIF) [file ppat.1012001.s002.tif]

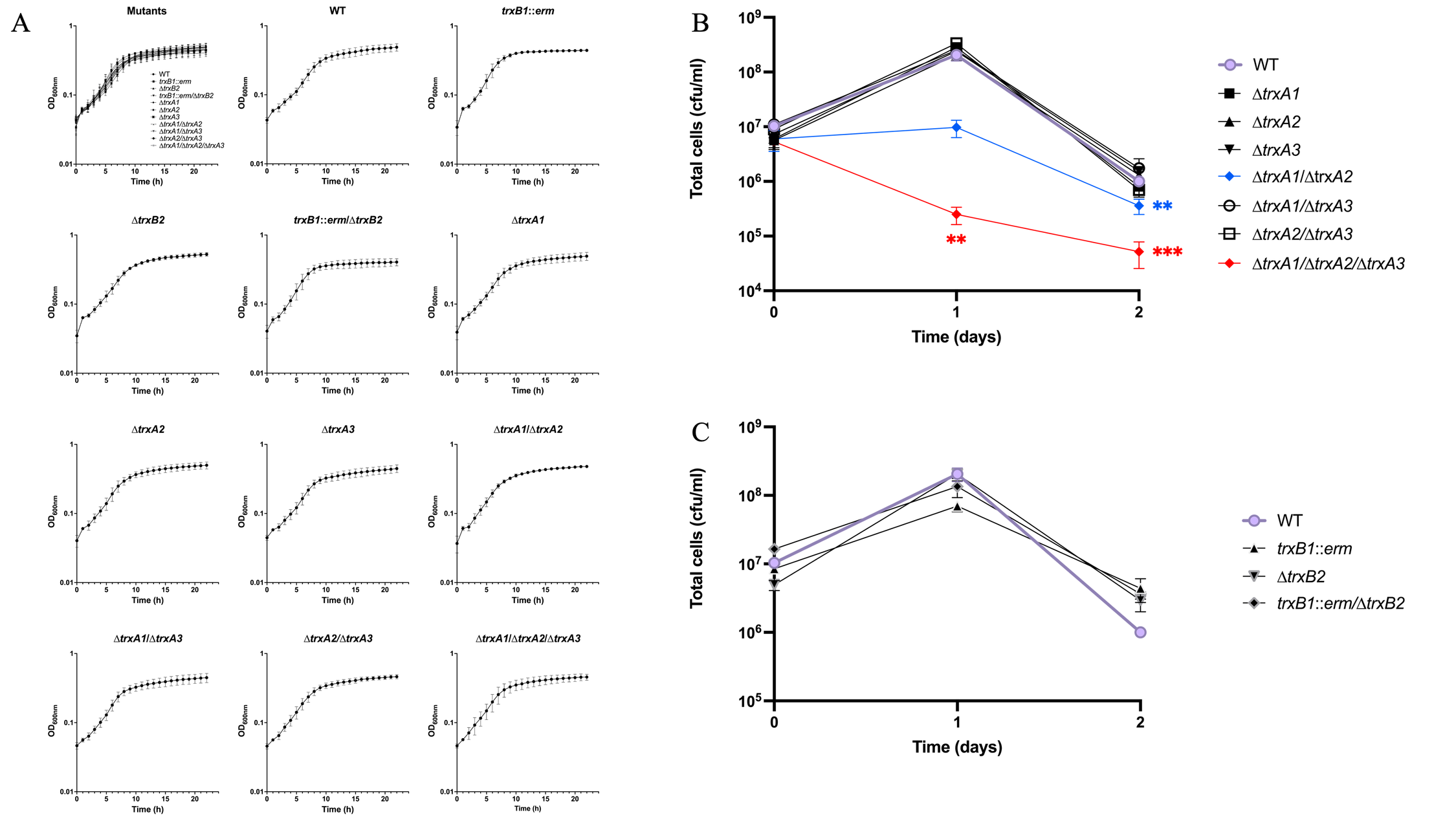

Supplement: S3 Fig — (A) Growth curves of the different trx mutants. Growth was monitored in a 96-well plate using an initial bacterial suspension at OD600nm 0.05 in TY for 24 h at 37°C. Experiments were performed in 5 biological replicates. Mean and SD are shown. The first panel presents all curves represented in other panels as individual curves. (B, C) Survival of (B) trxA and (C) trxB mutants. A bacterial suspension at OD600nm 0.05 in TY was prepared. Total bacteria were numerated daily over 2 days by plating serial dilutions on TY Tau plates. Experiments were performed in 5 biological replicates. Mean and SEM are shown. Multiple unpaired t-tests were performed. *: p-value<0.05, ** <0.01, *** <0.001. (TIF) [file ppat.1012001.s003.tif]

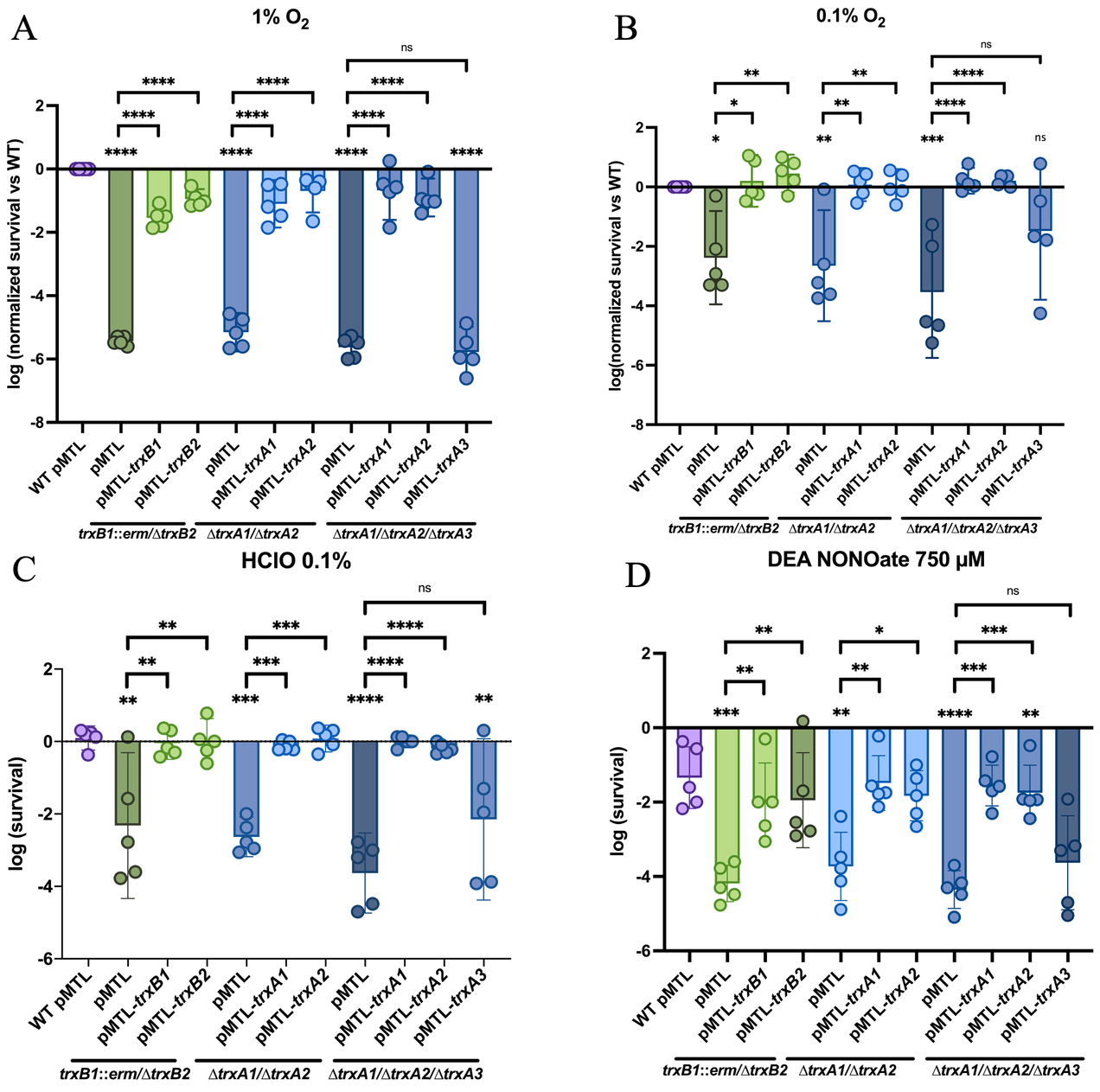

Supplement: S4 Fig — (A, B) Samples were serially diluted, plated in duplicate on TY Tau plates and incubated either in anaerobiosis or in hypoxia at (A) 1% O2 or (B) 0.1% O2 for 64 h. Survival was then normalized by doing the ratio of the mutant vs the WT. (C, D) Samples were serially diluted and plated on TY and on (C) TY + DEA NONOate 750 μM or (D) TY + HClO 0.1% and incubated for 24 h. Mean and SD are shown. Experiments were performed in 5 biological replicates. For all assays, one-way ANOVA were performed followed by Dunett’s multiple comparison tests. *: p-value<0.05, ** <0.01, *** <0.001 and **** <0.0001. (TIF) [file ppat.1012001.s004.tif]

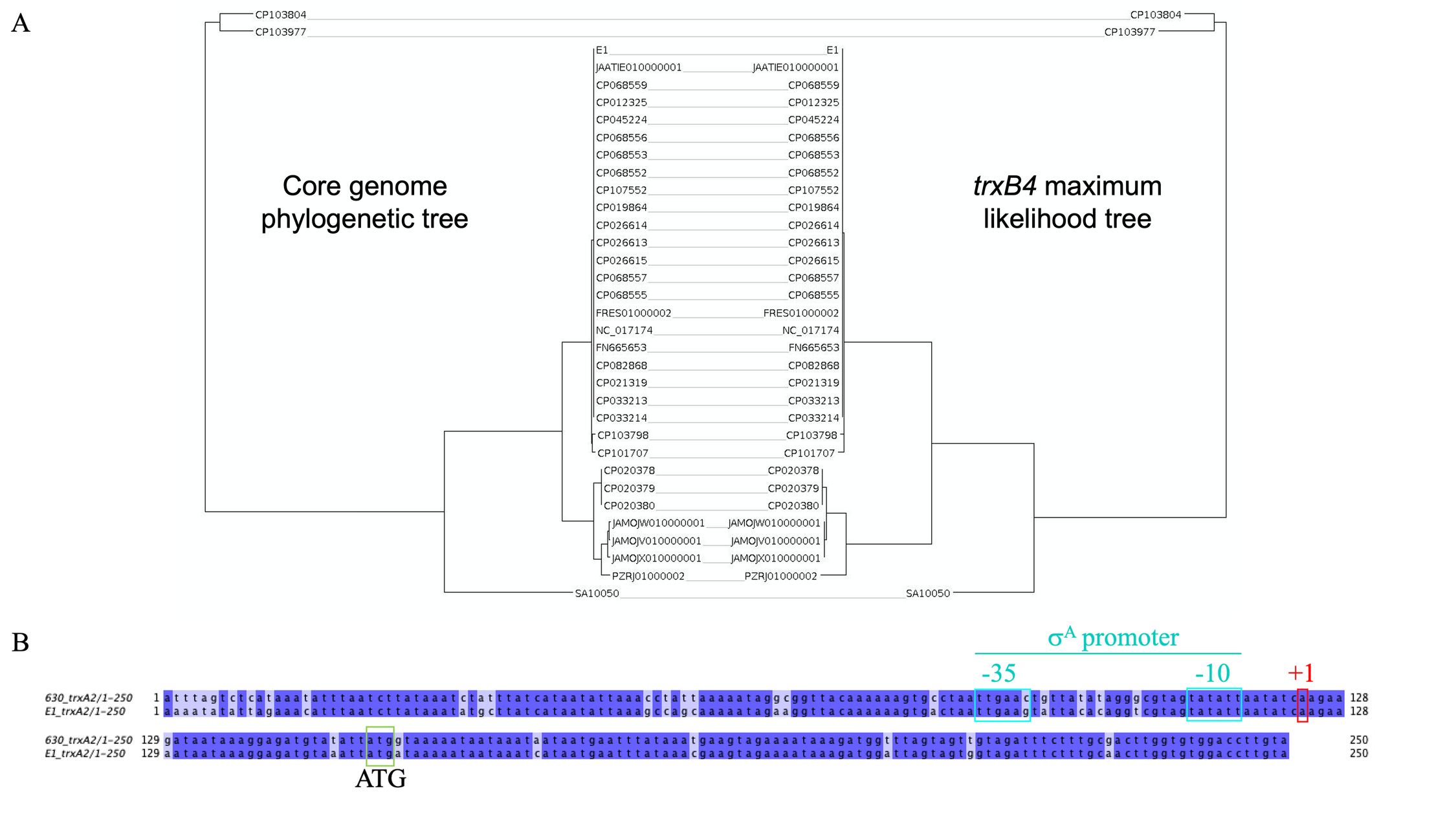

Supplement: S5 Fig — (A) Comparison of the C. difficile core genome and trxB4 evolution. The tanglegram representing the trxB4 tree (right) and the core-genome tree pruned to contain only genomes present in the trxB4 tree (left). The branch lengths are measured in substitutions per site and are on the same scale. The only difference between the two trees is that the trxB4 one is less resolved, which is explained by the limited phylogenetic signal in only one gene used for its reconstruction. The figure is produced with Dendroscope [111]. (B) Alignment of trxA2 promoter region in the 630Δerm and the E1 strains. Regions corresponding to the 150 bp upstream the trxA2 start codon (ATG) and the following 100 bp from the 630Δerm and the E1 strains were aligned using the MAFFT software [112]. The ATG, the TSS and the sA promoter [46] are indicated. Jalview software [113] was used for visualization. (TIF) [file ppat.1012001.s005.tif]

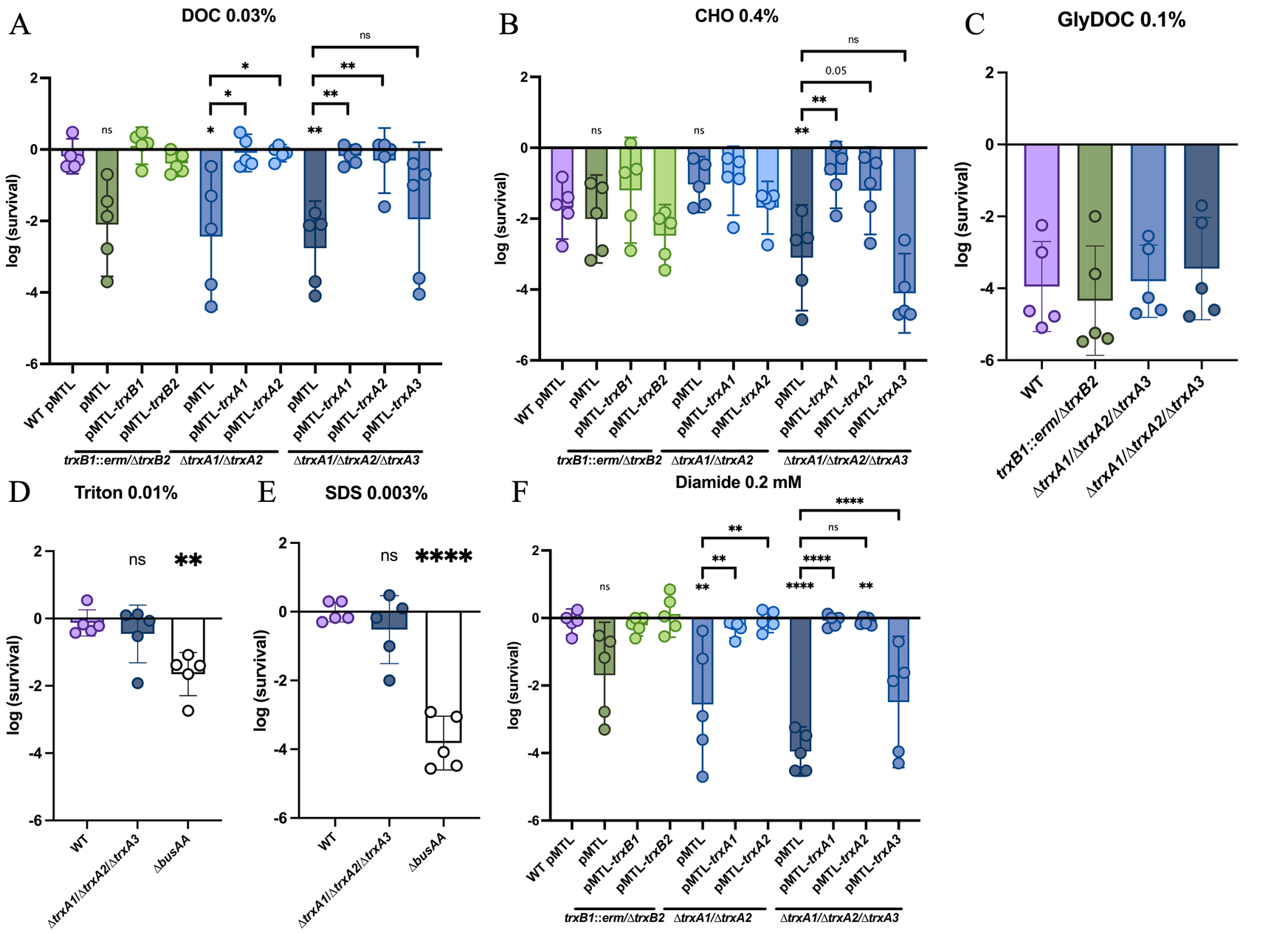

Supplement: S6 Fig — Strains were serially diluted and plated on TY and on (A) TY + DOC 0.03%, (B) TY + CHO 0.4%, (C) TY + GlyDOC 0.1%, (D) TY + Triton X-100 0.01%, (E) TY + SDS 0.003% or (F) TY + diamide 0.02% and incubated for 24 h. Survival was calculated by doing the ratio between CFUs in the last dilution with stress and CFUs in the last dilution without stress. Mean and SD are shown. Experiments were performed in 5 biological replicates. For all assays, one-way ANOVA were performed followed by Dunett’s multiple comparison test. *: p-value<0.05, ** <0.01, *** <0.001 and **** <0.0001. (TIF) [file ppat.1012001.s006.tif]

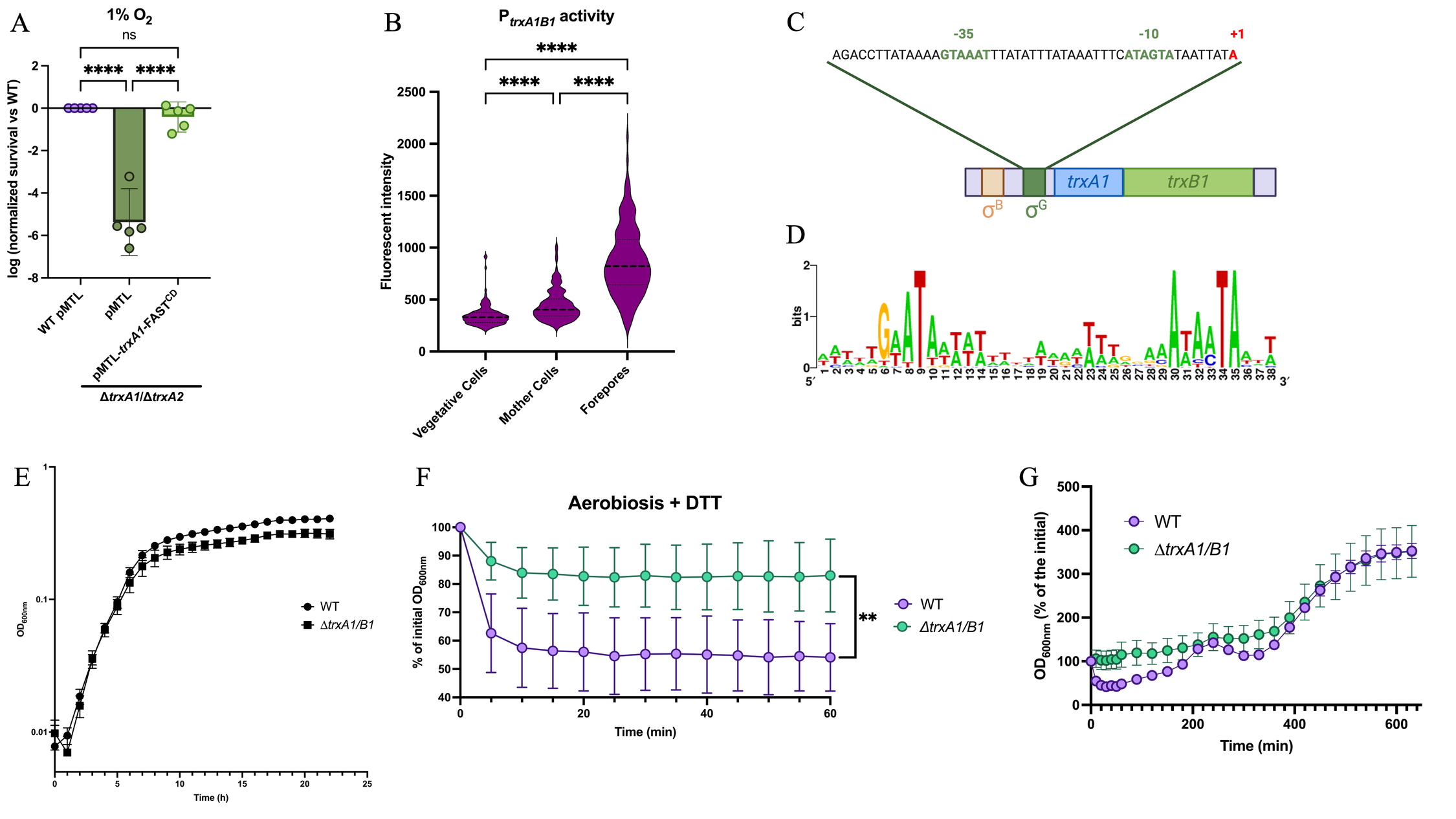

Supplement: S7 Fig — (A) Samples were serially diluted, plated in duplicate on TY Tau plates and incubated either in anaerobiosis or in hypoxia at 1% O2 for 64 h. Survival was then normalized by doing the ratio of the mutant vs the WT. Mean and SD are shown. Experiment was performed in 5 biological replicates. (B) Average PtrxA1B1-FASTCD fluorescence intensity of different compartments from acquired images of panel 7B. Each group consists in the measure of the average PtrxA1B1-FASTCD of 150 cells from two independent experiments. (C) Promoter identification through 5’RACE using RNA extracted from exponentially growing cells of strain 630Δerm sigB::erm. The TSS (+1) is indicated in red. Upstream this TSS, sB box is represented in orange, sG box in green. (D) sG consensus, Figure from [46]. (E) Growth curves of the ΔtrxA1/B1 mutants. Growth was monitored in a 96-well plate using an initial bacterial suspension at OD600nm 0.05 in TY for 24 h at 37°C. Experiments were performed in 5 biological replicates. Mean and SD are shown. (F) ~107 spores of WT strain and of ΔtrxA1/B1 mutant were exposed to 1% Tau to induce germination in air with 0.1% DTT. OD600nm was monitored every 5 min to evaluate germination. Experiments were performed in 5 replicates with at least 2 independent spore suspensions. Mean and SD are shown. (G) Outgrowth assay. ~107 spores of WT strain and of ΔtrxA1/B1 mutant were exposed to 1% Tau. OD600nm was monitored every 10 min for 1 h followed by every 30 minutes for 10 h. OD600nm was normalized by initial OD600nm. Experiments were performed in 5 replicates with at least 2 independent spore suspensions. Mean and SEM are shown. For O2-survival, one-way ANOVA was performed followed by Dunett’s multiple comparison tests. For fluorescence intensity quantification, Kruskal-Wallis tests were performed followed by Dunn’s multiple comparison test. For germination and outgrowth assay, two-way ANOVA were performed *: p-value<0.05, ** <0.01, *** <0.001 and **** <0.0001. (TIF) [file ppat.1012001.s007.tif]
