# Supplementary material for: The multiplicity of thioredoxin systems meets the specific lifestyles of Clostridia
Source: PLoS Pathog. 2024 Feb 8;20(2):e1012001. doi: 10.1371/journal.ppat.1012001 (PMC10880999; doi:10.1371/journal.ppat.1012001)
Supplement: S1 Table — (PDF) [file ppat.1012001.s008.pdf]

S1 Table. List of strains used in this study

Strain	Genotype	Origin
E. coli		
NEB10	*Δ(ara-leu) 7697 araD139 fhuA ΔlacX74 galK16 galE15 e14-* ϕ*80*d*lacZΔM15 recA1 relA1 endA1 nupG rpsL* (Str^R) *rph spoT1 Δ(mrr-hsdRMS-mcrBC)*	BioLabs
HB101(RP4)	*supE*44 *aa*14 *galK*2 *lacY*1 Δ(*gpt-proA*) 62 *rpsL*20 (Str^R)*xyl-5 mtl-1 recA*13 Δ(*mcrC-mrr*) *hsdS*B($r_B^- m_B^-$) RP4 (Tra^+ IncP Ap^R Km^R Tc^R)	Laboratory stock
C. difficile		
630Δ*erm*		Laboratory stock
E1		Laboratory stock
CDIP229	630Δ*erm sigB*::*erm*	[1]
CDIP529	630Δ*erm trxB1*::*erm*	This work
CDIP1456	630Δ*erm* pMTL84121	Laboratory stock
CDIP1461	630Δ*erm* Δ*trxB2*	This work
CDIP1486	630Δ*erm trxB1*::*erm* Δ*trxB2*	This work
CDIP1632	630Δ*erm trxB1*::*erm* Δ*trxB2* pMTL84121	This work
CDIP1636	630Δ*erm trxB1*::*erm* Δ*trxB2* pMTL84121-P-*trxB2*	This work
CDIP1640	630Δ*erm trxB1*::*erm* Δ*trxB2* pMTL84121-P-*trxB1*	This work
CDIP1796	630Δ*erm* Δ*trxA1B1*	This work
CDIP1797	630Δ*erm* Δ*trxA2*	This work
CDIP1811	630Δ*erm* Δ*trxA1*	This work
CDIP1812	630Δ*erm* Δ*trxA1* Δ*trxA2*	This work
CDIP1814	630Δ*erm* Δ*trxA1* Δ*trxA2* pMTL84121-P-*trxA1*	This work
CDIP1815	630Δ*erm* Δ*trxA1* Δ*trxA2* pMTL84121-P-*trxA2*	This work
CDIP1823	630Δ*erm* Δ*trxA1* Δ*trxA2* pMTL84121	This work
CDIP1884	630Δ*erm trxB1*::*erm* Δ*trxB2* pMTL84121-P-*trxB4* (E1 strain)	This work
CDIP1924	630Δ*erm* Δ*trxA3*	This work
CDIP1925	630Δ*erm* Δ*trxA1* Δ*trxA3*	This work
CDIP1933	630Δ*erm* Δ*trxA2* Δ*trxA3*	This work
CDIP1939	630Δ*erm* Δ*trxA1* Δ*trxA2* Δ*trxA3*	This work
CDIP1967	630Δ*erm* Δ*trxA1* Δ*trxA2* Δ*trxA3* pMTL84121	This work
CDIP1968	630Δ*erm* Δ*trxA1* Δ*trxA2* Δ*trxA3* pMTL84121-P-*trxA1*	This work
CDIP1969	630Δ*erm* Δ*trxA1* Δ*trxA2* Δ*trxA3* pMTL84121-P-*trxA3*	This work
CDIP1970	630Δ*erm* Δ*trxA1* Δ*trxA2* Δ*trxA3* pMTL84121-P-*trxA2*	This work
CDIP2113	630Δ*erm* Δ*grdAB*	This work
CDIP1982	630Δ*erm* pFT47-$P_{trxA1B1}$-$FAST^{CD}$	This work
CDIP1983	630Δ*erm sigB*::*erm* pFT47-$P_{trxA1B1}$-$FAST^{CD}$	This work
CDIP2157	630Δ*erm sigB*::*erm* pFT47-$FAST^{CD}$	This work
CDIP2075	630Δ*erm* pFT47-$P_{trxA1B1}$-TrxA1'-$FAST^{CD}$	This work
CDIP2203	630Δ*erm sigF*::*erm* pFT47-$P_{trxA1B1}$-$FAST^{CD}$	This work
CDIP2205	630Δ*erm sigG*::*erm* pFT47-$P_{trxA1B1}$-$FAST^{CD}$	This work
CDIP2234	630Δ*erm* Δ*trxA1* Δ*trxA2* pFT47-$P_{trxA1B1}$-TrxA1'-$FAST^{CD}$	This work

P: promoter, *erm*: erythromycin resistance gene. *trxA1 = CD1690*, *trxA2 = CD3033*, *trxA3 = CD2355, trxB1 = CD1691*, *trxB2 = CD2117*

1. Kint N, Janoir C, Monot M, Hoys S, Soutourina O, Dupuy B, et al. The alternative sigma factor σ^B plays a crucial role in adaptive strategies of *Clostridium difficile* during gut infection: Role of σ^B in Stress Adaptation in *C. difficile*. Environ Microbiol. 2017 May;19(5):1933–58.
