# Supplementary material for: The multiplicity of thioredoxin systems meets the specific lifestyles of Clostridia
Source: PLoS Pathog. 2024 Feb 8;20(2):e1012001. doi: 10.1371/journal.ppat.1012001 (PMC10880999; doi:10.1371/journal.ppat.1012001)
Supplement: S2 Table — (PDF) [file ppat.1012001.s009.pdf]

S2 Table List of plasmids

**Plasmid**	**Characteristics**	**Origin**
pMTL007	Plasmid for gene inactivation through Clostron	Laboratory stock
pMSR	Plasmid for gene deletion through allelic chromosomic exchange (ACE)	Laboratory stock
pMTL84121	Replicative plasmid in *E. coli* and *C. difficile* and conjugation-transmissible	Laboratory stock
pFT47	pMTL84121 derivative carrying the $SNAP^{CD}$ gene	[1]
pGEMTeasy	Plasmid for TA cloning for sequencing for 5'RACE	Promega
pDIA6190	pMTL007-Cdi-*trxB1-36a*	This work
pDIA6919	pMSR-ACE Δ*trxB2*	This work
pDIA7025	pMTL84121-P-*trxA1-trxB1*	This work
pDIA7042	pMTL84121-P-*trxB1*	This work
pDIA7050	pMTL84121-P-*trxB2*	This work
pDIA7108	pMSR-ACE Δ*trxA1-trxB1*	This work
pDIA7112	pMSR-ACE Δ*trxA2*	This work
pDIA7113	pMTL84121-P-*trxA2-trxB4* (strain E1)	This work
pDIA7118	pMSR-ACE Δ*trxA1*	This work
pDIA7122	pMTL84121-P-*trxA2*	This work
pDIA7129	pMTL84121-P-*trxA1*	This work
pDIA7156	pMTL74121-P-*trxB4* (strain E1)	This work
pDIA7162	pMTL84121-P-*grdX-trxB3-trxA3*	This work
pDIA7163	pMSR-ACE Δ*trxA3*	This work
pDIA7164	pMTL84121-P-*trxA3*	This work
pDIA7190	pFT47-$FAST^{CD}$	This work
pDIA7194	pFT47-$P_{trxA1B1}$-$FAST^{CD}$	This work
pDIA7236	pFT47-$P_{trxA1B1}$-*trxA1'*-$FAST^{CD}$	This work
pDIA7281	pMSR-ACE Δ*grdAB*	This work

ACE: allelic chromosomic exchange, P: promoter.

1. Pereira FC, Saujet L, Tomé AR, Serrano M, Monot M, Couture-Tosi E, et al. The Spore Differentiation Pathway in the Enteric Pathogen Clostridium difficile. PLoS Genet. 2013 Oct 3;9(10):e1003782.
