# Supplementary material for: The multiplicity of thioredoxin systems meets the specific lifestyles of Clostridia
Source: PLoS Pathog. 2024 Feb 8;20(2):e1012001. doi: 10.1371/journal.ppat.1012001 (PMC10880999; doi:10.1371/journal.ppat.1012001)
Supplement: S3 Table — (PDF) [file ppat.1012001.s010.pdf]

S3 Table. List of oligonucleotides

Primer	Sequence	Characteristics
EBSu	CGAAATTAGAAACTTGCGTTCAGTAAAC	Clostron *trxB1*
IMV690	AAAAAAGCTTATAATTATCCTTAGCTGGCCCACTGGTGCGCCCAGATAGGGTG	
IMV691	CAGATTGTACAAATGTGGTGATAACAGATAAGTCCCACTGCCTAACTTACCTTTCTTTGT	
IMV692	TGAACGCAAGTTTCTAATTTCGATTCCAGCTCGATAGAGGAAAGTGTCT	
IMV1113	TGGTCATGAGATTATCAAAAGGTTGGAACATAAAGACCACCATCATT	ACE mutagenesis *trxB2*
IMV1114	TCCAGCACCAATTACAATGATATCT	
IMV1115	AGATATCATTGTAATTGGTGCTGGAGGAGCTATTGCAGCCGTTCAA	
IMV1116	ATCGTAGAAATACGGTGTTTTTTGGAGAAATAGTTGTTATAGCTGGTA	
CA5	TGGTCATGAGATTATCAAAAGGGATGAGTTACTTCCAGATAGAAG	ACE mutagenesis *trxA1*
CA6	TTTTAAATCCTCCTATTAAACTTTATTATC	
CA7	GATAATAAAGTTTAATAGGAGGATTTAAAAGTATCTACAGTACCAACTATG	
CA19	ATCGTAGAAATACGGTGTTTTTTGTACTCTTAATTAGATTTTTCAACC	
CA9	TGGTCATGAGATTATCAAAAGGGCAGCAGGTCTTTATG	ACE mutagenesis *trxA2*
CA10	TTTCTTATTCCCCCTTGAAA	
CA11	TTTCAAGGGGGAATAAGAAAGGTCTTCCAACTATGGCT	
CA12	ATCGTAGAAATACGGTGTTTTTTGAAACACAGTTACCACTTAC	
CA1	TGGTCATGAGATTATCAAAAGGGTATTGAACTCCAATATATAAAATG	ACE mutagenesis *trxA3*
CA2	AATATACATCTCCTTTATTATCTTC	
CA3	GAAGATAATAAAGGAGATGTATATTGGAAAACCTGTAGATAGATTAAT	
CA4	ATCGTAGAAATACGGTGTTTTTTGTAATGTATCTCTCGTTACAG	
CA13	GATAATAAAGTTTAATAGGAGGATTTAAAAGCTGGTCAAGGG	ACE mutagenesis *trxA1*-*trxB1*
CA14	ATCGTAGAAATACGGTGTTTTTTGTAAACTTTTATTCCCATTCC	
AM007	TTTTTTGTTACCCTAAGTTTGGTTTATTACATGATACTTATGTATATG	ACE mutagenesis *grdAB*
AM008	TTTATTTTTCCTTTTTATTACTAAGTAAACTCATAATAATC	
AM009	AGTAATAAAAAGGAAAAATAAAAATAACACACACATATATTATATAG	
AM010	AGATTATCAAAAAGGAGTTTGGGCATTTAAGTTAACATC	
CM13	GATCGGTCTTGCCTTGCTC	Amplification of pMTL84121 for cloning through Gibson Assembly
IMV993	CTGGCGTTACCCAACTTAATCG	
IMV1183	CCGCTCGAGATATTGGAACTACATTTGAATTG	Amplification of P-*trxA1*-*trxB1*
IMV1151	GGGGATCCTTAAAAATAAAAAACTGTCTTGA	
IMV1200	AAAATGGGTGAGTATTATGAGA	Inverse PCR to obtain P-*trxB1*
IMV1201	CAGATGTATTTATAACTTTTGC	
IMV1331	GGACCACTGCCGATTATAGCT	Inverse PCR to obtain P-*trxA1*
CA24	GAGCAAGGCAAGACCGATCGGGAAAAAATGTCAACTATATTAAG	Amplification of P-*trxA2*
CA25	CGATTAAGTTGGGTAACGCCAGCATTAGCAATTAGACACTCATTTG	
IMV1202	GAGCAAGGCAAGACCGATCACAGCAGCATTAGTTTTGGTTAT	Amplification of P-*trxB2*
IMV1203	CGATTAAGTTGGGTAACGCCAGTGAACATGATAGATTAAGATATAAC	
IMV1235	GAGCAAGGCAAGACCGATCGAAAATACAAGAAATATGCAATATCAA	Amplification of P-*grdX*-*trxB3*-*trxA3*
CA43	CGATTAAGTTGGGTAACGCCAGCATAAAAACCTTTTTCTTCTTATCTTC	
IMV1300	TCTGCCTTGAATTCTTCTTTTACT	Inverse PCR to obtain P-*trxA3*
IMV1389	GGGAACTAGCAATCTGTAAATTAAG	
CA26	GAGCAAGGCAAGACCGATCGCTTATCATAATATTAAAGCCAG	Amplification of P-*trxA2*-*trxB4* (E1)
CA27	CGATTAAGTTGGGTAACGCCAGGAATCCACAATCAATAACAAC	
CA75	AATTTACATCTCCTTTATTATTTTC	Inverse PCR to obtain P-*trxB4*
CA76	TTAAAATAAATCATATATAAACAAAAAAC	
AAP	GGCCACGCGTCGACTAGTACGGGIIGGGIIGGGIIG	Abridged Anchor Primer
IMV710	TTATCATTACCAAACATGA	5’RACE mRNA amplification *trxB1*
IMV1209	GTTTCTACTGGTTTTCCATCTTT	5’RACE TA Cloning *trxB1*
IMV1156	CACCTGAAGAAGTTTTTACAAC	5’RACE mRNA amplification *trxB2*
IMV1157	TCAACACCTTGAGCAACTGC	5’RACE TA Cloning *trxB2*

IMV567	CTCCAAGTGCATTGGTTCCT	qPCR *trxB1*
IMV568	AATCTCCAGCAGCAAAGCAT	
IMV571	TCAAGCAGTTGCTCAAGGTG	qPCR *trxB2*
IMV572	AATTCTTGTTCCCCTGCACA	
IMV1239	TGGTTGTCCTGGAGAAAAAGA	qPCR *trxB3*
IMV1240	ATGCACTGTCTCCTCCACCT	
IMV1274	TTTCTTTGCGACTTGGTGTG	qPCR *trxA3*
IMV1275	GTCCACCTTCAAGAAGTTTGCT	
IMV1237	TATGAGCGATGAAGGGCATT	qPCR *CD3605.1*
IMV1238	AGCTGAAACTGGACATCCTTCT	
BD3	TTTTGTTGTGTCTATGAACCTTTGT	qPCR *gyrA*
BD4	TCCTTTACCAGCTCTTATTTGACTT	
BD9	GTTAAATGGGATAGAAGAGGTTGCT	qPCR *ccpA*
BD10	TATACCTTCCACTTGTTTTGCTCTC	
IMV1103	CCGAGCTCGAATTCGTAATCATGGT	Amplification of pFT47 for cloning through Gibson Assembly
IMV1140	CCGCTCGAGCATAAAATCATCCTCTCTTATATT	
IMV1105	ACCATGATTACGAATTCGAGCTCGGACATTTGAATTGTCAATACCA	Amplification of $P_{trxA1B1}$
IMV1443	CATGGTATTTTCCTCCTTTCTCCAGATGTATTTATAACTTTTGC	
IMV1499	AACCAAAGGCTACATGCTCCATTAAATATGATTTTACTTGCATTTCAATT TT	Amplification of $P_{trxA1B1}$-*trxA1’*
