# Supplementary material for: The multiplicity of thioredoxin systems meets the specific lifestyles of Clostridia
Source: PLoS Pathog. 2024 Feb 8;20(2):e1012001. doi: 10.1371/journal.ppat.1012001 (PMC10880999; doi:10.1371/journal.ppat.1012001)
Supplement: Attachment — Submitted filename: Answer to reviewers.pdf [file ppat.1012001.s014.pdf]

Answer to reviewers

Part I - Summary

Reviewer #1: The manuscript by Anjou et al. provides important new insight into the functions of thioredoxin systems in bacteria. By conducting a thorough phylogenetic analysis of the conservation and composition of the thioredoxin (Trx) thiol and protein repair pathway, the authors demonstrate that many members of the Firmicutes are distinguished by encoding multiple Trx system. In contrast, most bacteria encode only a single system thioredoxin reductase (TrxB), which may act on multiple thioredoxins (TrxA). TrxAs reduce disulfide bonds on oxidized proteins, while TrxB recycles TrxA soluble mediators.

To assess whether the multiple Trx systems encoded in C. difficile have differential functions or are simply functionally redundant, the authors systematically analyzed the function and requirement for the three strictly conserved Trx systems found in strain 630. Using genetic analyses involving single, double, and triple mutants and complementation strains, they show that the Trx1 and Trx2 systems are critical for providing resistance against low concentrations of oxygen, reactive nitrogen species, bleach, and hydrogen peroxide. This is consistent with these systems being regulated by the stress response sigma factor, SigB. They further demonstrate that these Trx systems help C. difficile to resist bile acids but not other detergents like SDS or Triton, suggesting that bile acids specifically induce unwanted disulfide bonds. Consistent with this hypothesis, the addition of the reducing agent DTT reversed the toxicity of the bile acid tested, providing critical new insight into how bile acids cause toxicity in C. difficile.

While the Trx1 and Trx2 systems help *C. difficile* vegetative cells resist oxygen and additional small molecule stresses, the authors' data indicate that the Trx3 system promotes sporulation potentially by impacting the levels of the glycine reductase complex, which trxA3 is encoded within. Despite this finding, it is unclear whether the Trx3 system is found within the spore. In contrast, TrxA1 and TrxB1 have been found in the spore proteome, and the authors show that the transcription of these genes is specifically upregulated in the forespore in a SigG-dependent manner. Excitingly, they show that the TrxA1/B1 appears to affect spore resistance to bleach, suggesting that this system may be functional in dormant spores. The authors present germination data suggesting that a Δ*trxA1-trxA2* deletion mutant does not germinate as fully in ambient air vs. during anaerobiosis, but this difference is quite subtle.

Finally, the authors also identify a fourth Trx system encoded in some strains of C. difficile and show that the Trx4 system can functionally complement a triple mutant strain lacking the Trx1-3 systems for O2 tolerance.

In summary, this nicely written and designed study provides novel insights into the Trx system in C. difficile, revealing a functional specialization for the multiple systems during different parts of C. difficile's lifecycle and reveals the impact of bile acids on inducing disulfide bond stress.

Reviewer #2: This manuscript by Anjou et al. identifies and characterizes the thioredoxin systems encoded by the anaerobic pathogen C. difficile. The authors generate a series of mutants in the trxA and trxB mutants, as well as double mutants and a triple mutant, to demonstrate their thioredoxin functions and roles in tolerance to oxidative stress. The manuscript is well-written, the data are clearly presented, and the conclusions are supported

by the results. The findings represent a significant advance in the understanding of oxidative stress responses by this pathogen. Minor suggestions are listed below.

Reviewer #3: The manuscript by Anjou and co-authors reports has several points in its favor. It reports on an analysis of the thioredoxin systems present across bacteria, which highlights the multiplicity of these systems in the Clostridia and then focuses on the genetically tractable human pathogen C. difficile, a strict anaerobe, to dissect their function. The findings are important in the context of infection by this organism but also of broader importance. Specifically, that at least two systems, TrxA1 and TrxA2, are important for survival in the presence of O2 and that the TrxA systems and TrxB1/B2 are important for survival in the presence of molecules that are produced during inflammation; that TrxA1/TrxB1 influence spore germination and hypochlorite resistance; that TrxA3 contributes to sporulation via a GrdAB-dependent mechanism; and the elegant observation that the Trx systems are involved in coping with the ability of bile salts to induce disulfide-bond formation.
The experimental work is carefully done. Overall, the manuscript adds important information to our knowledge on the biology of C. difficile.

Part II – Major Issues: Key Experiments Required for Acceptance

Reviewer #1: Since different bile acids have been shown to have differential effects on C. difficile's physiology, e.g. deoxycholate can induce biofilm formation, it would be helpful to test an additional bile acid such as lithocholate to see if it also causes disulfide bond stress, or if the authors' finding is more specific to deoxycholate.

We tested the impact of lithocholate on the survival of the 630Δ*erm* strain, the *trxA1trxA2*, the *trxB1-trxB2* and the triple *trxA* mutants. At the limit of lithocholate solubility (2% in DMSO and 0.1% final concentration in the plate), we observe no difference in survival of the double *trxB* and *trxA* mutants and the triple *trxA* mutant at a concentration, which is 3-fold higher than that of DOC. We thus used glycodeoxycholate, a secondary bile acid, for which we were able to demonstrate an inhibitory effect. We add a Fig. S6C to indicate the impact of glycodeoxycholate to *trx* mutant survival. However, we found no difference between the WT strain and multi mutants, suggesting that the disulfide bond formation activity of glycodeoxycholate is reduced compared to the one with DOC or CHO. In addition, line 329 we modify the title of the paragraph to "Two Trx systems are required to cope with disulfide-bond formation induced by some bile salts". We also add a sentence line 345 to indicate. "Interestingly, this phenotype was not seen when glycodeoxycholate (GlyDOC) was used (Fig. S6C), suggesting a specific action of CHO and DOC." We also modified the conclusion (line 372) to indicate that "Conjointly, these results show the importance of Trx systems in the repair of disulfide-bonds induced by some bile acids, CHO and DOC."

Since the optical density assay measures changes in spore density across the population, the difference could be an effect across the entire population or a subset of spores that never germinate. Visualizing the germination of WT vs. ΔtrxA1-trxA2 mutants using phase-contrast microscopy at a few of the timepoints (and fixing the spores at a given timepoint) would provide useful insight into whether the Trx1 system impacts germination. A more convincing phenotype may be during the outgrowth phase, where ΔtrxA1-trxA2 spores would likely be killed more readily during outgrowth when exposed to 1% O2 or ambient air during germination in the presence of taurocholate and media).

We assessed a germination assay in aerobiosis under the microscope, imaging germinating spore after exposure of 0, 20 and 180 min to 1% Taurocholate. This experiment showed that a subpopulation of Δ*trxA1/B1* spores were not switching from bright phase to dark phase after 3 hours. This subpopulation decreases at 180 min compared to 20 min, suggesting that these spores are still viable but take more time to germinate in aerobiosis. We added the results in Fig. 8C-D. We added in the text (line 477) "We also evaluated the germination in aerobiosis after 20 and 180 min under the microscope (Fig. 8D-E). After 20 min, all the spores of the WT strain were phase-dark, while a subpopulation of approximately 15% of spores of the Δ*trxA1/B1* mutant remained phase-bright. After 180 min, this subpopulation only represented 5% of spores, which was still significantly higher than the WT strain." We then performed an outgrowth assay with the WT strain and the *trxA1B1* mutant. We performed several tests. We failed to observe regrowth of cells following germination in aerobiosis after 24 h. This absence of growth was also observed when the spores of the WT and trxA1B1 strains are exposed to air for germination (1 h) and then put again in anaerobiosis. We then performed outgrowth/restart of growth during 10 h in anaerobiosis as previously described (Mooyutu et al., 2017). Under these conditions, we did not observe a difference between the WT and Δ*trxA1B1* strains. We added this result in Fig. S7G and referred to it line 486 "We also performed an outgrowth assay in anaerobiosis of the mutant but observed no difference with the WT strain (Fig. S7G). However, as this assay requires anaerobiosis to observe growth, we are not able to conclude if the germination defect observed in aerobiosis would lead to an outgrowth defect in similar conditions." This result confirms that the germinating *trxA1B1* spores have a problem to face oxidative stress rather than a strict germination defect.

Reviewer #2: n/a

Reviewer #3: It is my opinion that two experiments would add value to the manuscript, one is related to spore germination; it has several components but in reality is a single experiment. I think the extent of spore germination could be affected in a trxA1/B1 mutant because every abundant spore surface proteins are cysteine-rich. Disulfide-bond formation could thus affect spore structure and indirectly germination. If so, DTT could revert the defect (or a mutant in the gene for one of the most abundant of those proteins). Since germination is triggered by taurocholic acid, this would provide a link to the author´s finding that the thioredoxin systems are involved with coping with the formation of disulphide bonds promoted by bile acids.
The other, less important, is to look at expression of trxA3 (which is involved in sporulation) during sporulation, using the FAST reporter.
From my review letter:
1) Germination occurs without macromolecular synthesis and so it is difficult to envisage how the activity of trxA1/B1 could influence the extent of germination. Is it possible that the difference seen in the presence of oxygen is related to some structural/mechanical effect on the proteins that form the spore surface? Several of the proteins that compose the exosporium are cysteine rich and disulfide bond formation (perhaps promoted by the presence of taurocholic acid?) could perhaps induce a structural alteration of the spore surface that would interfere with the extent of germination. If so, could the addition of DTT restore the extent of spore germination to the trxA1/B1 mutant? Alternatively, a mutant in the gene coding for the most abundant of these cysteine-rich proteins, cdeM, could also eliminate differences between the wt and the trxA1/B1 mutant.

We tested the germination of spores of both the WT strain and the *trxA1/B1* mutant in aerobiosis in presence of DTT (Fig. S7E). The difference is still significative between the two strains, suggesting that DTT doesn't restore the phenotype. This result suggests that the

reduction of disulfide bond of the Trx system during germination does not explain alone the phenotype observed. This phenotype could be rather explained by the need of a Trx system during spore formation leading to a phenotype during germination. Alternatively, as the Trx systems are known to be electron donor for other proteins, the lack of this system induces a loss of function of at least one target protein important for germination. We now added a supplementary Fig S7E to present the data of germination in the presence of air and DTT. We also modified the text to add the following sentences (line 481). "In addition, the reduced decrease of OD_{600nm} observed in aerobiosis for the Δ*trxA1*/*B1* was still observed when DTT was added (Fig. S7F). This result suggests that the reduced efficiency of germination is not due only to the disulfide bond reduction activity of Trx systems. Moreover, as a phenotype was only visible in aerobiosis, it seems that this germination defect is due to an increased sensibility to oxidative stress."

Since the germination assay was done in BHI, it should in principle measure both the initial rate of germination (decrease in the OD600) but also the outgrowth of cells from germinated spores (increase in the OD600) after a certain period of time, presumably in excess of 60 min, which is the last time point examine. Do the authors have data past this point? Are there differences in outgrowth among strains/conditions?

We performed an outgrowth assay with the WT strain and the *trxA1B1* mutant. We performed several tests. We failed to observe regrowth of cells following germination in aerobiosis after 24 h. This absence of growth was also observed when the spores of the WT and *trxA1B1* strains are exposed to air for germination (1 h) and then put again in anaerobiosis. We then performed outgrowth/restart of growth during 10 h in anaerobiosis as previously described (Mooyutu et al., 2017). Under these conditions, we did not observe a difference between the WT and Δ*trxA1B1* strains. We added this result in Figure in Fig. S7G, and referred to it line 486 "We also performed an outgrowth assay in anaerobiosis of the mutant but observed no difference with the WT strain (Fig. S7G). However, as this assay requires anaerobiosis to observe growth, we are not able to conclude if the germination defect observed in aerobiosis would lead to an outgrowth defect in similar conditions." This result confirms that the germinating *trxA1B1* spores have a problem to face oxidative stress rather than a strict germination defect.

2) Expression of *trxA3* is under the control of a sA-type promoter. But how is expression during sporulation? whole sporangium, increased in the forespore or in the mother cell. Have the authors looked at trxA3 expression using the FAST system?

We obtained a fusion between the promoter region of *trxA3* and the FAST gene, but the expression of this fusion remains under the threshold of detection during vegetative growth phase and sporulation. In a recent proteome comparing the proteome of the 630Δ*erm* strain in exponential growth phase and in stationary phase (16 h), six proteins encoded by the grd operon are more produced overnight (GrdD, GrdA, TrxA3, TrxB3 and GrdC, GrdE) (Lacotte and Martin-Verstraete, unpublished). The *grd* operon is also positively controlled by SigH 5-10 fold strongly suggesting an involvement in stationary phase and sporulation (Saujet et al, 2011). The Grd proteins are not detected in the proteome of the spore (Lawley, 2019). However, TrxB3 encoded by the *grd* operon is detected associated with the spore surface (Abyankhar et al, J. proteome Res, 2013; Diaz-GonZalez et al, J. Proteomic, 2015). This suggests that the operon is probably not expressed in the forespore but rather in the mother cell during sporulation.

Part III – Minor Issues: Editorial and Data Presentation Modifications

Reviewer #1: For the different mutants that they analyze, please include the "delta" Δ symbol to indicate that it is a gene deletion. The graphs can be tricky to interpret when just referring to the gene that has been deleted. We corrected the graphs and the text to indicate the type of mutation.

Please provide molar concentrations for the bile acids tested We added the molar concentrations to the method sections, line 789.

Line 101: the statement that primary bile acids allow spore germination is not technically correct because chenodeoxycholate is a primary bile acid that is a potent inhibitor of germination.
We modify the text line 101 to indicate that most of primary bile acids allow spore germination
Line 105: the statement as written makes it seem like the switch to glucose fermentation leads to an increase in oxygen, but the use of the term "hypoxia" makes it seem like oxygen is scarce and that is what leads to barrier dysfunction. My understanding was that butyrate consumes oxygen, resulting in a more hypoxic environment that is better for gut barrier function? We modified the text to make clearer the increase of O_2 due to the depletion of butyrate-producing bacteria : "This switch, from the oxidation of butyrate towards glucose fermentation that consumes less O_2, increases the O_2-tension in the gut contributing to a decrease of the integrity of the intestinal barrier."

Line 198: Since this is the first report of the use of the FAST fluorogen reporter in C. difficile to my knowledge, it would be helpful to explain more how the reporter works. The ability of the reporter to be used under strictly anaerobic conditions could be emphasized here.
We now added the following sentence in the text "The FAST system is O_2 independent and functions both in anaerobiosis and in the presence of O_2. This system has been used successfully in *Clostridium acetobutylicum* (49)" We added a reference by Streett HE, Kalis KM, Papoutsakis ET. Appl Environ Microbiol. 2019 Jul;85(14):e00622-19.

Line 220: Have the authors looked under the microscope at their cells during later stages of the bile acid treatment? It is striking that they don't see the optical density decrease even though they see a 2-log decrease in CFUs.
The data in FigS3 have been done in TY in the absence of bile acid. For the triple mutant, we observed a decreased in OD_{600nm} at 48h. Our data suggests that even if the OD is still not different at 24h, the viability already dropped but maybe the cell lysis started after 24h.

Regarding Fig 2E, the authors mention that they previously demonstrated that SigB activity is heterogeneous, with some cells expressing the SigB-dependent transcriptional reporter and others not. However, in their graph quantifying the fluorescence of their PtrxA1-FAST reporter during exposure to 1% O2 in a WT background, they don't see a proportion of cells that are off (i.e. around 100 fluorescence units) in contrast with a qualitative analysis of the fluorescence micrographs of the cells shown in Fig 2D. Could the authors comment on the discrepancy?
The heterogeneity of expression has been published (Kint et al, Environmental Microbiol, 2019 and Kint et al mBio, 2020) with other SigB targets (*revrbr1*, *revrbr2*, *fdpF* and *fdpA*)

and with SNAP fusions instead of a FAST fusion, which is likely less sensitive that the SNAP system based on an enzymatic reaction. In addition, the fusions in the previous work were tested in the absence of a stress. Following exposure to 1% O_2, the global level of expression of the fusion increases and maybe less cells were below the threshold leading to a more homogeneous fluorescence intensity. However, we still observed a few cells with a high level of fluorescence while most of the cells remains less fluorescent.

Fig 8B and 8C should use a two-way ANOVA to compare the difference between the curves rather than individual data points, since the assay involves repeated measurements on the same samples rather than discrete measurements on multiple samples. We performed the two-way ANOVA and corrected the representation, the legend and the methods section.

Well written manuscript but a few small typos or grammatical errors to be corrected:

Lines 25, 26: remove the hyphens after "thiol-" and "protein-" and "thioredoxin-"
We have done

Line 51: please modify the sentence so that it reads "The thioredoxin (Trx) system plays a crucial. We have done

Line 54: please add a hyphen so that it reads "clostridial-specific". We added a hyphen.

Line 60: and Line 97: please delete "an". We remove "an".

Line 112: the phrase "and the dispersion of newly-formed spores in the environment" seems out of place and could likely be deleted. We deleted the sentence.

Line 214: please replace "but" with "except" We replace but by except in the sentence.

Line 235: please add "the" before "O2-level" We added the in the sentence.

Line 242: "simple" should be "single". We replace simple by single mutants.

Line 335: "phenotypical" could be "phenotypic". We replace phenotypical by phenotypic.

Line 369-372: these sentences should be written in the past tense. We modify the tense.

Line 402: please consider replacing "actually" with "interestingly". We have done as suggested.

Reviewer #2: The comment in the title "meets the specific needs of Clostridia" is vague. Presumably there are several trx in these anaerobes in order to support the greater need to combat oxidative stresses that they encounter?
As suggested by the reviewer, we slightly modify the title "The multiplicity of Thioredoxin systems meets the specific lifestyles of Clostridia", assuming that the multiplicity of Trx is not only due to the high level of oxidative stress present in the life cycle of Clostridia, but also to specific functions beyond the oxidative stress response (spore-formation, Stickland metabolism).

Ln 95: the Stickland reactions also occur outside of the Clostridia. We replaced "clostridial-

specific metabolic pathways such as Stickland reactions" by "metabolic pathways such as Stickland reactions present in proteolytic clostridia".

Ln 115: There may be systems besides the Trx that lack similarity to known mechanisms and have not yet been identified.
We modified the text to indicate "*C. difficile*, like most Clostridia, lacks synthesis pathways and reductases associated with other known thiol repair systems, *e.g.*, glutathione, mycothiol and bacillithiol". We also modified the last sentence to indicate that "Even if an uncharacterized thiol repair system may exist, the absence of these conventional alternative systems suggests a crucial role of Trx systems in *C. difficile* physiology".

The abbreviations NTR and FFTR should be defined.
Line 82 we indicated that NAD(P)H-using Trx Reductase or NTR for NADPH Trx Reductase.
line 88 for ferredoxin-dependent flavin Trx Reductase (FFTRs). We have added flavin in the text.
Ln 152: manipulatable? We have modified

Ln 158: Please change "indicating" to suggests, unless there is evidence of ferredoxin cofactor. We modify the sentence to indicate that "By contrast, TrxB1 lacks these NAD(P)H binding motifs and clusters with other clostridial FFTRs, suggesting that this copy uses ferredoxin as cofactor"

Reviewer #3: Several minor points related to the text and the figures, taken from my review letter.

Abstract:
Line 24: on their cysteines We have modified the text.
Lines 45-47: suggestion: "…most likely meets specific need off the Clostria in adaptation to strong stresses sporulation and Stickland pathways" We have modified the text as suggested.
Author summary:
Lines 62-64: suggestion: the needs of cells during growth and differentiation, not only in Clostridia but perhaps in other multiple-Trx-reductase… We have modified the text as suggested.
Introduction:
Line 78: oxidized cysteines in proteins. We have modified.

Line 85: Fe-S clusters. We have modified.
Line 101: preventing host colonization by this organism. We have modified the text as suggested.
Line 105: "increases the O2 tension in the gut" and "resulting hypoxia" is not very clear. We modified the text to make clearer the increase of O2 due to the depletion of butyrate-producing bacteria : "This switch, from the oxidation of butyrate towards glucose fermentation that consumes less O_2, increases the O_2-tension in the gut contributing to a decrease of the integrity of the intestinal barrier."
Line 121: the life cycle of C. difficile and their associated regulations. We modified
Results:
Line 138/139: in the latter two groups. We have modified.
Fig 1: line 146: circles and squares. We have modified.

Fig 2, line 190: cells instead of bacteria. We have modified
Line 193: As C. difficile faces. We have modified
Line 200: induced in the presence. We have modified
Line 201: fluorescent signal in the presence of O2. We have modified
Line 204: In summary, the different trx loci are differentially regulated. We have modified the sentence as suggested
Line 219: or multiple mutants. We have modified
Line 235: the. survival in the presence of 1% O2 We have modified
Line 248: the same results were obtained in. the presence of 0.1% of O2 We have modified
Fig. 3, line 251: Tau could be defined here. We added the definition
Fig. 3, line 254: then normalized as the ratio of We have modified
Fig. 3, line 254: Samples (not strains) withdrawn from cultures were serially diluted… We have modified here and at the other locations in the text
Line 281: widespread instead of spread? We have modified
Line 293: several independent gene loss events We have modified
Fig. 4, line 302: strains serially diluted? We have modified here and at the other locations in the text
Line 321: title, suggestion: Two Trx systems are required to cope with disulfide-bond formation induced by bile salts. We have modified the title of the paragraph as suggested by the reviewer.
Fig. 5, line 339: strains serially diluted? We replace strains by samples.
Lines 369-370: why was sporulation evaluated using ethanol shock and not heat resistance tests? Also in the methods section. The ethanol shock is more reproducible in our hands than heat stress.
Line 374: …slight. Contribution of the two other TrxAs to sporulation when TrxA3 is absent, or in the absence of TrxA3.We replace "to this mechanism in absence of TrxA3" by "to sporulation in the absence of TrxA3".
Line 390: since the grdAB genes are in the middle of the operon, it would be helpful here if the nature of the mutation used is specified; is it an in-frame deletion or are polar effects to be expected? The Δ*grdAB* mutant, which has been obtained by the ACE system, is an in-frame deletion of the two genes. We keep the 8 first codon of *grdA* and the last two of *grdB*. We added in the supplementary table S3 the oligonucleotides used for the construction of the pMSR derivative allowing inactivation of *grdAB*.
Line 403: a reference to the FAST reporter and its use could be included here that mentions its use in anaerobes, if there is one. References are given in the methods section but their inclusion here would facilitate reading. The FAST reporter system has been used for the first time in an anaerobe, *Clostridium acetobutylicum,* in 2019 (Streett HE et al, Applied environmental Microbiol 85 (14)e00622-19). We added this reference in the text line 202-203 when we use the FAST system for the first time.
It is also important to state whether the trxA1-FAST fusion is functional. Will trxA1-FAST complement a double trxA1/trxB2 mutant, for example, for growth on plates upon exposure to 1%O2?
We expressed the pMTL84121-*trxA1*-FAST fusion in the *trxA1/trxA2* double mutant and performed a survival at 1% O_2. We were able to show that the translational fusion complements the phenotype, indicating that the TrxA1-FAST protein fusion is functional. We added the Figure in Fig S7A, and mentioned it in the text line 416 "We confirmed that this protein fusion was able to complement the Δ*trxA1*/Δ*trxA2* mutant in a O_2-survival experiment (Fig. S7A)."
Line 404: what is the percentage of vegetative cells, mother cells and forespores in which accumulation of the *trxA1*-FAST fusion is seen? Same for the transcriptional fusion, just

below. In the tested conditions, we observed fluorescence in 100% of forespore of sporulating cells for the translational fusion strongly suggesting the production of TrxA1 in forespore.
Line 407: expression of $P_{trxA1B1}$-FAST increased in the forespore in terms of percentage or signal intensity or both? Quantification of these parameters seems important here because it would allow a correlation of the time/intensity of expression with stages in morphogenesis.
We quantified the expression in the vegetative cells, in mother cells and in forespores. We showed that the fluorescence intensity was increased in the mother cell compared to the vegetative cell, and that the expression was increased in the forespore compared to the mother cell. The quantification plot was added in Fig. S7B and referred at line 424.
Line 410-411: why expression of the transcriptional fusion is no longer detected in phase bright forespores? Looking at the images included in Fig 7 (panels B and C), was phase contrast microscopy used or other contrast technique?
Phase contract microscopy was used in the paper. Concerning the absence of signal in the phase bright forespore, we are not able to say if the FAST reagent is able to penetrate the mature spore. In another experiment where free mature spores from the same construction were observed, no signal was detected in the free spores. No data have been published for the use of the FAST reporter in spore.
Figure 7C: cartoons on the right; sK is represented as active in the mother cell, together with sE and sB in the “mature forespore” cartoon, but not in the “complete engulfment” cartoon. Why? We modified to indicate that SigK is active at the stage complete engulfment.
Also, in the bottom set of panels, sK is not represented in the sigG mutant, but there is at least some activity of sK in this mutant. SigK has been added.
Please check scale bars in panels A and B (10 µm) and C (3 µm). We checked the scales, and they are the good ones.
Line 444: the text mentions the consensus sequence of a promoter recognized by sG; Fig. 7D should be referred to here; panel D of Fig. 7 could also represent the accepted consensus sequence for sG-recognized promoters.
In a supplementary Figure S7C-D (indicated line 458), we have now added the consensus published in the paper of Soutourina et al, 2020 (Frontiers in Microbiology, Front Microbiol. 2020 Aug 13;11:1939. doi: 10.3389/fmicb.2020.01939).
Line 450: I would prefer “mutant blocked following engulfment completion” as engulfment is a process, not a stage We have modified
Line 456: unclear why the trxA1/B1 system would delay sporulation. Can the authors comment on this?
We hypothesized that the *trxA1B1* mutant might grow faster than the WT strain and thus trigger sporulation earlier. However, by performing growth curve we observed no difference. We added the curve in Fig S7E and added in the text line 469: “This quicker sporulation is not due to a difference in growth (Fig. S7E).” Our current data did not explain this increase in spore percentage at day 1.
Lines 460-461: “significantly” lower reduction in OD600 suggesting a “slightly” lower efficiency of germination do not seem compatible. We deleted slightly.
Line 466: more impacted than the one of…. We have modified.
Line 479: calculating instead of doing. We now indicate “Survival was estimated by calculating the ratio of CFUs between treated and non-treated spores”
Methods
Line 626: what is pMSR? The pMSR plasmid is a plasmid developed in our laboratory allowing allele chromosomic exchange. This plasmid, which have been published in reference 92, uses a toxin of a Toxin/antitoxin system expressed under the control of a Ptet inducible promoter to force the second crossing-over events as indicated line 658. The reference 92 was

added again when we use this plasmid and this plasmid is indicated with the associated reference in the TableS2 in the supplementary material.
Line 635: the expected deletion is an in-frame deletion? Yes, the expected deletion is in frame, no antibiotic resistance cassette has been introduced in the inactivated genes.
Line 641: include here a reference to the table listing the primers used. This has been done.
Line 693: rhodamine. This was modified
Line 696: what is pFT47? pFT47 is a pMTL84121 derivative carrying the $SNAP^{CD}$ gene. This plasmid has been added in the TableS2. This plasmid has been published in Pereira et al, Plos Genet, 2013, 9(10):e1003782. This reference has been added line 727.
Line 710: after 24 h of growth in anaerobiosis. We added of growth to the sentence.
Line 739: sporulation rate was estimated by calculating the ration of spores to total cells over time. The sentence was modified as suggested.
Line 755: dilutions of cultures at midlog? We indicated now: "Dilutions of a suspension at an OD_{600nm} of 0.5"
Line 744: spores were enumerated. We modified.
Line 814: using the Auto Strategy function? We now indicated "using the Auto Strategy option"
References:
Reference 52 is not complete. We complete the reference as requested.
FIGURES
Figure 2A: the trxA2/trxB4 operon, found in some strains, could also be represented.
This has been added in Fig 2A.
Figure 2D: please check the scale bar. In panel E, the 600 ms does not need to be included in the title of the y axis. The scale bars have been checked and are correct and we removed the 600 ms from the y axis.
Figure 6: perhaps % of sporulation is better as the title for the y axis of all panels. We modified the title of the y axis.
Figure. 7: see comments above on phase contrast, scale bars, sG consensus and cartoons on the right of panel C. Also, the data on expression of trxA1/B1 is split between two figures (Figure 2 and Figure 7). It would be probably better to include the data in a single figure or in consecutive figures, but this may be difficult to do given the present organization of the manuscript. Comments about phase contrast, scale bars, sigG consensus and cartoons have been addressed above. Regarding the organization of the paper, we were not able to merge Fig 2 and Fig 7. The two figures correspond to independent parts of the paper, and we thus believe that it is easier to follow with the two figures.
Figure 8, panel B: "air" in the top panels could be replaced by aerobic conditions or equivalent. Air was replaced by Aerobiosis.
