# Supplementary material for: The multiplicity of thioredoxin systems meets the specific lifestyles of Clostridia
Source: PLoS Pathog. 2024 Feb 8;20(2):e1012001. doi: 10.1371/journal.ppat.1012001 (PMC10880999; doi:10.1371/journal.ppat.1012001)
Supplement: Attachment — Submitted filename: Response to Reviewers.docx [file ppat.1012001.s012.docx]

Response to Reviewers

- The abstract type has been modified in accordance with the PLOS Pathogens format.
