# Supplementary material for: The multiplicity of thioredoxin systems meets the specific lifestyles of Clostridia
Source: PLoS Pathog. 2024 Feb 8;20(2):e1012001. doi: 10.1371/journal.ppat.1012001 (PMC10880999; doi:10.1371/journal.ppat.1012001)
Supplement: Attachment — Submitted filename: Review of Anjou et al.docx [file ppat.1012001.s013.docx]

The manuscript by Anjou and co-authors reports on the functional characterization of the various thioredoxin systems found in the human pathogen C. difficile, a strict anaerobe. The authors also provide an analysis of the thioredoxin systems present across bacteria, which highlights the complexity of these systems in the Clostridia. The functional analysis reported is elegant, in particular the fact that some experiments were conducted under anaerobiosis and in the presence of a defined level of O2. Several findings are really important: i) that at least two systems, TrxA1 and TrxA2, are important for survival in the presence of O2 and that the TrxA systems and TrxB1/B2 are important for survival in the presence of molecules that are produced during inflammation; ii) the finding of a role for trxA1/TrxB1 in spore germination and hypochlorite resistance; iii) the discovery that TrxA3 contributes to sporulation via a GrdAB-dependent mechanism; iv) the conclusion that the Trx systems are involved in coping with the ability of bile salts to induce disulfide-bond formation.

The manuscript is overall very clear, and the experimental work is very sound. This work was due.

Two points:

Germination occurs without macromolecular synthesis and so it is difficult to envisage how the activity of trxA1/B1 could influence the extent of germination. Is it possible that the difference seen in the presence of oxygen is related to some structural/mechanical effect on the proteins that form the spore surface? Several of the proteins that compose the exosporium are cysteine rich and disulfide bond formation (perhaps promoted by the presence of taurocholic acid?) could perhaps induce a structural alteration of the spore surface that would interfere with the extent of germination. If so, could the addition of DTT restore the extent of spore germination to the trxA1/B1 mutant? Alternatively, a mutant in the gene coding for the most abundant of these cysteine-rich proteins, cdeM, could also eliminate differences between the wt and the trxA1/B1 mutant.

Since the germination assay was done in BHI, it should in principle measure both the initial rate of germination (decrease in the OD600) but also the outgrowth of cells from germinated spores (increase in the OD600) after a certain period of time, presumably in excess of 60 min, which is the last time point examine. Do the authors have data past this point? Are there differences in outgrowth among strains/conditions?

Expression of trxA3 is under the control of a sA-type promoter. But how is expression during sporulation? whole sporangium, increased in the forespore or in the mother cell. Have the authors looked at trxA3 expression using the FAST system?

Additional points:

Abstract:

Line 24: on their cysteines

Lines 45-47: suggestion: “…most likely meets specific need off the Clostria in adaptation to strong stresses sporulation and Stickland pathways”

Author summary:

Lines 62-64: suggestion: the needs of cells during growth and differentiation, not only in Clostridia but perhaps in other multiple-Trx-reductase…

Introduction:

Line 78: oxidized cysteines in proteins

Line 85: Fe-S clusters

Line 101: preventing host colonization by this organism

Line 105: “increases the O2 tension in the gut” and “resulting hypoxia” is not very clear.

Line 121: the life cycle of C. difficile and their associated regulations.

Results:

Line 138: in the latter two groups

Fig 1: line 146: circles and squares

Fig 2, line 190: cells instead of bacteria

Line 193: As C. difficile faces

Line 200: induced in the presence

Line 201: fluorescent signal in the presence of O2

Line 204: In summary, the different trx loci are differentially regulated

Line 219: or multiple mutants

Line 235: the. survival in the presence of 1% O2

Line 248: the same results were obtained in. the presence of 0.1% of O2

Fig. 3, line 251: Tau could be defined here.

Fig. 3, line 254: then normalized as the ratio of

Fig. 3, line 254: Samples (not strains) withdrawn from cultures were serially diluted…

Line 281: widespread instead of spread?

Line 293: several independent gene loss events

Fig. 4, line 302: strains serially diluted?

Line 321: title, suggestion: Two Trx systems are required to cope with disulfide-bond formation induced by bile salts

Fig. 5, line 339: strains serially diluted?

Lines 369-370: why was sporulation evaluated using ethanol shock and not heat resistance tests? Also in the methods section.

Line 374: …slight. Contribution of the two other TrxAs to sporulation when TrxA3 is absent, or in the absence of TrxA3.

Line 390: since the grdAB genes are in the middle of the operon, it would be helpful here if the nature of the mutation used is specified; is it an in-frame deletion or are polar effects to be expected?

Line 403: a reference to the FAST reporter and its use could be included here that mentions its use in anaerobes, if there is one. References are given in the methods section but their inclusion here would facilitate reading. It is also important to state whether the trxA1-FAST fusion is functional. Will *trxA1*-FAST complement a double trxA1/trxB2 mutant, for example, for growth on plates upon exposure to 1%O2?

Line 404: what is the percentage of vegetative cells, mother cells and forespores in which accumulation of the trxA1-FAST fusion is seen? Same for the transcriptional fusion, just below.

Line 407: expression of PtrxA1B1-FAST increased in the forespore in terms of percentage or signal intensity or both? Quantification of these parameters seems important here because it would allow a correlation of the time/intensity of expression with stages in morphogenesis.

Line 410-411: why expression of the transcriptional fusion is no longer detected in phase bright forespores? Looking at the images included in Fig 7 (panels B and C), was phase contrast microscopy used or other contrast technique?

Figure 7C: cartoons on the right; sK is represented as active in the mother cell, together with sE and sB in the “mature forespore” cartoon, but not in the “complete engulfment” cartoon. Why? Also, in the bottom set of panels, sK is not represented in the sigG mutant, but there is at least some activity of sK in this mutant.

Please check scale bars in panels A and B (10 µm) and C (3 µm).

Line 444: the text mentions the consensus sequence of a promoter recognized by sG; Fig. 7D should be referred to here; panel D of Fig. 7 could also represent the accepted consensus sequence for sG-recognized promoters.

Line 450: I would prefer “mutant blocked following engulfment completion” as engulfment is a process, not a stage (also in other places throughout the text).

Line 456: unclear why the trxA1/B1 system would delay sporulation. Can the authors comment on this?

Lines 460-461: “significantly” lower reduction in OD600 suggesting a “slightly” lower efficiency of germination do not seem compatible.

Line 466: more impacted than the one of…

Line 479: calculating instead of doing.

Methods

Line 626: what is pMSR?

Line 635: the expected deletion is an in-frame deletion?

Line 641: include here a reference to the table listing the primers used.

Line 693: rhodamine.

Line 696: what is pFT47?

Line 710: after 24 h of growth in anaerobiosis

Line 739: sporulation rate was estimated by calculating the ration of spores to total cells over time.

Line 755: dilutions of cultures at midlog?

Line 744: spores were enumerated

Line 814: using the Auto Strategy function?

References:

Reference 52 is not complete.

FIGURES

Figure 2A: the trxA2/trxB4 operon, found in some strains, could also be represented.

Figure 2D: please check the scale bar. In panel E, the 600 ms does not need to be included in the title of the y axis.

Figure 6: perhaps % of sporulation is better as the title for the y axis of all panels.

Figure. 7: see comments above on phase contrast, scale bars, sG consensus and cartoons on the right of panel C. Also, the data on expression of trxA1/B1 is split between two figures (Figure 2 and Figure 7). It would be probably better to include the data in a single figure or in consecutive figures, but this may be difficult to do given the present organization of the manuscript.

Figure 8, panel B: “air” in the top panels could be replaced by aerobic conditions or equivalent.
